# Clinical characteristics and BGA-optimized pretest probability of pulmonary embolism in the elderly

**DOI:** 10.1007/s00063-024-01235-8

**Published:** 2025-01-22

**Authors:** T. Pätz, K. Gruber, S. Kupp, G.-M. Schmidtke, A. Fürschke, F. Sayk, T. Stiermaier, I. Eitel, S. Wolfrum, M. Meusel

**Affiliations:** 1 University Heart Center Lübeck, Department of Cardiology, Angiology and Intensive Care Medicine, University of Lübeck, German Center for Cardiovascular Research (DZHK), partner site Hamburg/Kiel/Lübeck, Ratzeburger Allee 160, 23538 Lübeck, Germany; 2 Department of Radiology and Nuclear Medicine, University Hospital of Schleswig Holstein, Campus Lübeck, Ratzeburger Allee 160, 23538 Lübeck, Germany; 3 Department of Internal Medicine I, University of Lübeck, Ratzeburger Allee 160, 23538 Lübeck, Germany; 4 Emergency Department, University Hospital Schleswig-Holstein, Campus Lübeck, Ratzeburger Allee 160, 23538 Lübeck, Germany

**Keywords:** Wells criteria, Computed tomography pulmonary angiography, D‑Dimer, Blood gas analysis, Elderly, Wells-Kriterien, Computertomographische pulmonalarterielle Angiographie, D‑Dimer, Blutgasanalyse, Ältere

## Abstract

**Background:**

Pulmonary arterial embolism (PE) is not well characterized in elderly patients. In addition, unnecessary computed tomography pulmonary angiography (CTPA) examinations are often performed within this patient group, especially if the pretest probability is low.

**Objective:**

To identify differences in clinical presentation in patients aged ≥80 years compared to patients <80 years and the effect of a BGA-optimized pretest probability to reduce unnecessary CTPAs according to age category.

**Materials and methods:**

A retrospective analysis of patients with suspected PE and subsequent CTPA was performed, with evaluation of clinical data including capillary blood gas analysis (BGA) parameters (including standardized partial pressure of oxygen [sPaO_2_]) over a 5-year period. Subsequently, the clinical characteristics of patients with confirmed PE were compared between the two age groups. In addition, an age-adjusted analysis of a BGA-optimized algorithm was performed in patients with a low pretest probability (PTP) according to the Wells score to reduce unnecessary CTPAs.

**Results:**

PE was confirmed in 433 of 1538 patients with suspected PE, of which *n* = 98 (22.6%) were ≥ 80 years of age. Elderly patients with PE were less frequently male (*p* < 0.001), had lower rates of tachycardia (*p* = 0.021), but higher rates of cardiovascular disease history (*p* = 0.001) and oxygen administration at admission (*p* = 0.006) compared to those < 80 years. Signs of right heart dysfunction (*p* = 0.047) and elevated cardiac biomarkers (troponin: *p* < 0.001; nt-pro-BNP: *p* = 0.026) were also more common in the elderly. Additionally, simplified Pulmonary Embolism Severity Index (sPESI, *p* < 0.001) and in-hospital or 30-day death risk (*p* < 0.001) were higher in the elderly. Using a BGA-optimized algorithm with sPaO_2_, unnecessary CTPA examinations were reduced by 33.2% in younger patients (75 of 226 without PE) and 23.5% in elderly patients (8 of 34 without PE).

**Conclusions:**

Elderly patients with PE are characterized by higher clinical risk markers and elevated mortality rates compared to younger patients. In patients with suspected PE but low PTP, however, a significant number of unnecessary CTPAs could be avoided by using an BGA-optimized pretest algorithm in elderly patients.

**Supplementary Information:**

The online version of this article (10.1007/s00063-024-01235-8) contains supplementary material, which is available to authorized users.

## Abstract (de)

**Hintergrund:**

Die Lungenarterienembolie (PE) ist bei älteren Patienten nicht gut untersucht. Darüber hinaus werden in dieser Patientengruppe häufig unnötige computertomographische pulmonalarterielle Angiographien (CTPA) durchgeführt, insbesondere bei geringer Vortestwahrscheinlichkeit (PTP).

**Ziel der Arbeit:**

Ermittlung von Unterschieden in der klinischen Präsentation bei Patienten im Alter von ≥80 Jahren im Vergleich zu Patienten <80 Jahren sowie Evaluation einer Blutgas(BGA)-optimierten Vortestwahrscheinlichkeit in Abhängigkeit der Altersgruppe.

**Material und Methode:**

Über einen Zeitraum von 5 Jahren erfolgte eine retrospektive Analyse von Patienten mit Verdacht auf PE und anschließender (CTPA), wobei klinische Daten sowie Parameter der kapillaren Blutgasanalyse (kurz BGA, inklusive des standardisierten Sauerstoffpartialdrucks [spaO_2_]) ausgewertet wurden. Anschließend wurden klinische Merkmale der Patienten mit bestätigter PE zwischen den beiden Altersgruppen verglichen. Darüber hinaus wurde die altersadjustierte Analyse eines BGA-optimierten Algorithmus bei Patienten mit niedriger PTP gemäß Wells-Score durchgeführt, um unnötige CTPA zu reduzieren.

**Ergebnisse:**

Eine PE wurde bei 433 von 1538 Patienten mit entsprechendem Verdacht bestätigt. Davon waren 98 Patienten (22,6 %) ≥ 80 Jahre alt. Ältere Patienten mit PE waren seltener männlich (*p* < 0,001) und hatten seltener Tachykardien (*p* = 0,021), aber häufiger kardiovaskulären Erkrankungen in der Vorgeschichte (*p* = 0,001) sowie eine höhere Sauerstoffgabe bei Aufnahme (*p* = 0,006) als Patienten < 80 Jahre. Anzeichen einer Rechtsherzdysfunktion (*p* = 0,047) und erhöhte kardiale Biomarker (Troponin: *p* < 0,001; nt-proBNP: *p* = 0,026) waren ebenfalls häufiger bei älteren Menschen zu finden. Darüber hinaus waren der simplified Pulmonary Embolism Severity Index (sPESI, *p* < 0,001) und das Risiko, im Krankenhaus oder nach 30 Tagen zu sterben, bei älteren Patienten höher (*p* < 0,001). Durch die Anwendung eines BGA-optimierten Algorithmus in Kombination mit spaO_2_ konnten unnötige CTPA bei jüngeren Patienten (75 von 226 ohne PE) um 33,2 % und bei älteren Patienten (8 von 34 ohne PE) um 23,5 % reduziert werden.

**Diskussion:**

Ältere Patienten mit PE besitzen im Vergleich zu jüngeren Patienten höhere klinische Risikomarker und eine höhere Sterblichkeitsrate. Bei älteren Patienten mit Verdacht auf PE, aber niedriger PTP, könnte unter Verwendung eines BGA-optimierten Vortestalgorithmus eine beträchtliche Anzahl unnötiger CTPA vermieden werden.

## Introduction

As a result of demographic changes with increased life expectancy and further improvements in medical care, the average age of emergency care patients is expected to increase and people aged ≥ 80 years will represent an increasing proportion of patients [1]. Therefore, these patients should receive special consideration in studies. However, it is notable that older patients tend to be underrepresented in clinical trials [2].

The prevalence of pulmonary artery embolism (PE) correlates positively with age [3]. Hence, due to demographic changes, more patients with suspected PE are expected to present to the emergency department (ED). As part of the diagnostic process, it is recommended that a pretest probability (PTP) is determined prior to computed tomography pulmonary angiography (CTPA) with, for example, the Wells representing a well-established diagnostic tool, regardless of age [4]. Using the two-stage classification, PE can be expected in about 8.4% of cases in the “PE unlikely” category and in 34.4% in the “PE likely” category [5]. However, it is problematic that particularly in the group of patients with low PTP, only a few patients actually will have confirmed PE [6–8], leading to frequent unnecessary CTPA in this patient group. Efforts should therefore be made to reduce unnecessary CTPA in primary assessment. In addition to the PTP, a blood gas analysis (BGA) is regularly performed in clinical practice, and although it does not have a specific role in determining PTP [4] it often influences further diagnostics. In a previous publication [9], we were able to show that a significant number of CTPA can be avoided by combining the Wells score with standardized PaO_2_ as a calculated product of the BGA even with increased D‑dimer threshold without missing a pulmonary artery embolism. However, as the physiological aspects of the lungs also change with increasing age [10], the effectiveness of this modified algorithm in elderly patients remains unclear. Therefore, the objective of this study was to evaluate the clinical presentations of patients with pulmonary artery embolism stratified by age, as well as to analyze blood gases and a BGA-optimized PTP in elderly patients with suspected pulmonary artery embolism.

## Materials and methods

### Study design and patients

**Fig. 1:**
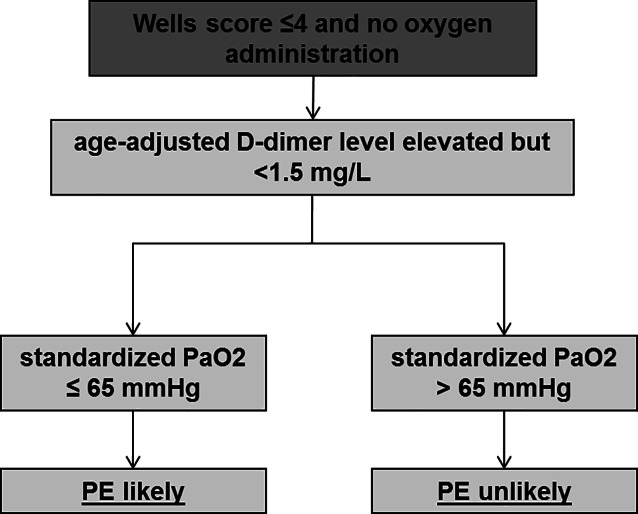
Workflow for patients with suspected PE but low pretest probability and available capillary blood gas analysis. PE pulmonary embolism, standardized PaO_2_ partial pressure of oxygen standardized to a PaCO_2_ of 40 mm Hg [9]

This study is a subanalysis of the previously published EMBOLISM study [9]. In brief, the aim of this single-center retrospective trial was to optimize PTP in the group of patients with a low PTP according to the Wells score (Wells score ≤ 4) using BGA. The hospital’s standard procedure for patients with suspected PE includes an assessment of the PTP using the Wells score and, if necessary, a D-dimer test (D-dimer Hemosil HS 500 with the ACL Analyser Top 750, Werfen, Germany), whereby an age-adjusted cut-off value was used for patients over 50 years of age. A total of 1538 patients were identified over a period of 5 years. All patients underwent a CTPA examination. Of these, 433 patients suffered a PE. Using a retrospectively calculated standardized PaO (standardized PaO_2_ = PaO_2_ − 1.66 × (40 − PaCO_2_)), a new algorithm was developed (Fig. 1), which allowed for raising the D‑dimer cut-off to < 1.5 mg/L. This resulted in an overall reduction of 31.9% of CTPA examinations without missing one PE [9]. The study was approved by the local ethical committee (reference number: 19-318A).

In the current study, the cohort of patients with confirmed PE was divided into elderly patients defined as ≥ 80 years and patients < 80 years. Subsequently, both clinical and diagnostic variables of these patient groups were analyzed in patients with confirmed PE. In addition, an age-related analysis of the capillary blood gases was performed. Based on the original study, the effectiveness of the previously published algorithm was analyzed by age group. In this process, only patients with low PTP (≤ 4 points) according to the Wells criteria [11–13] and capillary BGA were considered. In order to exclude the effect of oxygen therapy on the calculation of the standardized PaO_2_, patients with oxygen application were not included in the new algorithm.

### CT pulmonary angiography

The CTPA was carried out using a 128-detector Siemens Somatom Definition AS+ and AS scanner (Siemens Healthineers, Erlangen, Germany). The scan covered the area from the clavicles to the diaphragm in the craniocaudal direction, with a detector scan area of up to 800 mm. A layer thickness of 1 mm was selected for the reconstruction, and the tube current and voltage were 100 mAs and 120 kV, respectively, with CARE Dose used for quality reference. A low osmolar nonionic contrast medium (100 ml) was injected through a peripheral venous catheter at a rate of 5 ml/s.

### Statistical analysis

The statistical analysis was conducted using IBM SPSS Statistics 29.0 (Armonk, NY, USA), considering a two-sided *p*-value of less than 0.05 as statistically significant. Categorical variables were assessed with either the Chi-squared test or Fisher’s exact test, and their results are reported as numbers and percentages. Continuous variables were evaluated using the Mann–Whitney U test and are presented as medians with interquartile ranges (IQR). Baseline characteristics and BGA parameters were compared between patients < 80 years and those ≥ 80 years of age with confirmed PE. Scatter plots with linear regression graphs were used to display the results of the GBA.

## Results

### Baseline characteristics of patients with confirmed PE

During the study period, PE was confirmed in 433 of 1538 patients, with *n* = 98 (22.6%) aged ≥ 80 years and *n* = 335 (77.4%) aged < 80 years. Table 1 shows the detailed baseline characteristics of the two subgroups. Patients aged ≥ 80 years were less frequently male (*p* < 0.001), had a lower prevalence of tachycardia (*p* = 0.021), and had more frequently a history of cardiovascular disease (*p* = 0.001). Moreover, more patients aged ≥ 80 years were more likely to received oxygen administration (*p* = 0.006) compared to patients aged < 80 years. Right heart dysfunction in CTPA or echocardiography (*p* = 0.047) and elevated troponin levels (*p* < 0.001) were more frequent in older patients. In addition, sPESI (*p* < 0.001) and an intermediate–high mortality risk (*p* = 0.004), as well as the risk of in-hospital death (*p* < 0.001), were higher in elderly patients, while a low risk of in-hospital or 30-day death was more frequent in patients aged < 80 years (*p* < 0.001).

**Table 1:** Baseline characteristics and risk stratification of patients with confirmed pulmonary embolism (PE)

	< 80 years (*n* = 335)	≥ 80 years (*n* = 98)	*p*-value
*Male*	175 (52.2)	29 (29.6)	***p*** **≤** **0.001**
*Age*	64 (52, 74)	84 (82, 87)	–
*Central PE*	155 (46.3)	49 (50.0)	*p* = 0.515
*Segmental PE*	141 (42.1)	42 (42.9)	*p* = 0.908
*Subsegmental PE*	39 (11.6)	7 (7.1)	*p* = 0.264
*Chest pain*	150 (44.8)	40 (40.8)	*p* = 0.491
*Dyspnea*	250 (74.6)	70 (71.4)	*p* = 0.601
*Hemoptysis*	14 (4.2)	2 (2.0)	*p* = 0.386
*Syncope*	36 (10.7)	16 (16.3)	*p* = 0.157
*Clinical signs of deep vein thrombosis*	90 (26.9)	27 (27.6)	*P* = 0.898
*Tachycardia (>* *100* *bpm)*	121 (36.1)	23 (23.5)	***p*** **=** **0.021**
*S1Q3 pattern*	47 (14.0)	9 (9.2)	*p* = 0.235
*T wave inversion in V1–V4*	44 (13.1)	19 (19.4)	*p* = 0.142
*Complete/incomplete RBBB*	45 (13.4)	17 (17.3)	*p* = 0.412
*History of cardiovascular disease*	58 (17.3)	32 (32.7)	***p*** **=** **0.001**
*History of pulmonary disease*	71 (21.2)	17 (17.3)	*p* = 0.476
*History of renal disease*	22 (6.6)	10 (10.2)	*p* = 0.217
*Active cancer*	67 (20.0)	13 (13.2)	*p* = 0.141
*Previous PE or DVT*	92 (27.5)	24 (24.5)	*p* = 0.606
*D‑dimer*^*a*^*, mg/L*	4.46 (2.07, 9.55)	5.54 (2.81, 11.36)	*p* = 0.129
*Administration of oxygen*^*b*^			***p*** **=** **0.006**
Unknow	23 (6.9)	2 (2.1)	
Yes	73 (21.8)	35 (36.1)	
No	239 (71.3)	60 (61.9)	
*Oxygen flow rate in liters per minute*^*c*^	4 (2, 4)	4 (2, 8)	*p* = 0.148
*Right heart dysfunction in CTPA or echocardiography*^*d*^	132 (39.6)	50 (51.5)	***p*** **=** **0.047**
*Elevated Troponin serum concentration*^*e*^	172 (56.8)	78 (83.9)	***p*** **<** **0.001**
*Elevated NTpro-BNP serum concentration*^*f*^	84 (42.6)	35 (59.3)	***p*** **=** **0.026**
*sPESI*	1 (0, 1)	1 (1, 2)	***p*** **<** **0.001**
*EMR: low risk*^*g*^	95 (28.4)	2 (2.1)	***p*** **<** **0.001**
*EMR: intermediate low risk*^*h*^	138 (41.3)	49 (50.0)	*p* = 0.133
*EMR: intermediate high risk*^*h*^	87 (26.0)	41 (41.8)	***p*** **=** **0.004**
*EMR: high risk*^*h*^	12 (3.6)	6 (6.1)	*p* = 0.387
*Confirmed DVT*^*i*^	171 (54.3)	55 (61.1)	*p* = 0.280
*In-hospital death*^*j*^	6 (1.8)	9 (9.2)	***p*** **=** **0.001**

Data presented as *n*/*N* (%) or median (IQR). Numbers in bold type indicate a significant difference

*PE* pulmonary embolism, *DVT* deep vein thrombosis, *RBBB* right bundle branch block, *BGA* blood gas analysis, *CTPA* computed tomography pulmonary angiography, *sPESI* simplified PESI (Pulmonary Embolism Severity Index), *EMR* early mortality risk of in-hospital or 30-day death

^a^*n* = 416, ^b^*n* = 432, ^c^*n* = 101, ^d^*n* = 430, ^e^*n* = 396, ^f^*n* = 256, ^g^*n* = 430, ^h^*n* = 431, ^i^*n* = 405, ^j^*n* = 432

### Blood gas analysis and pretest probability

**Fig. 2:**
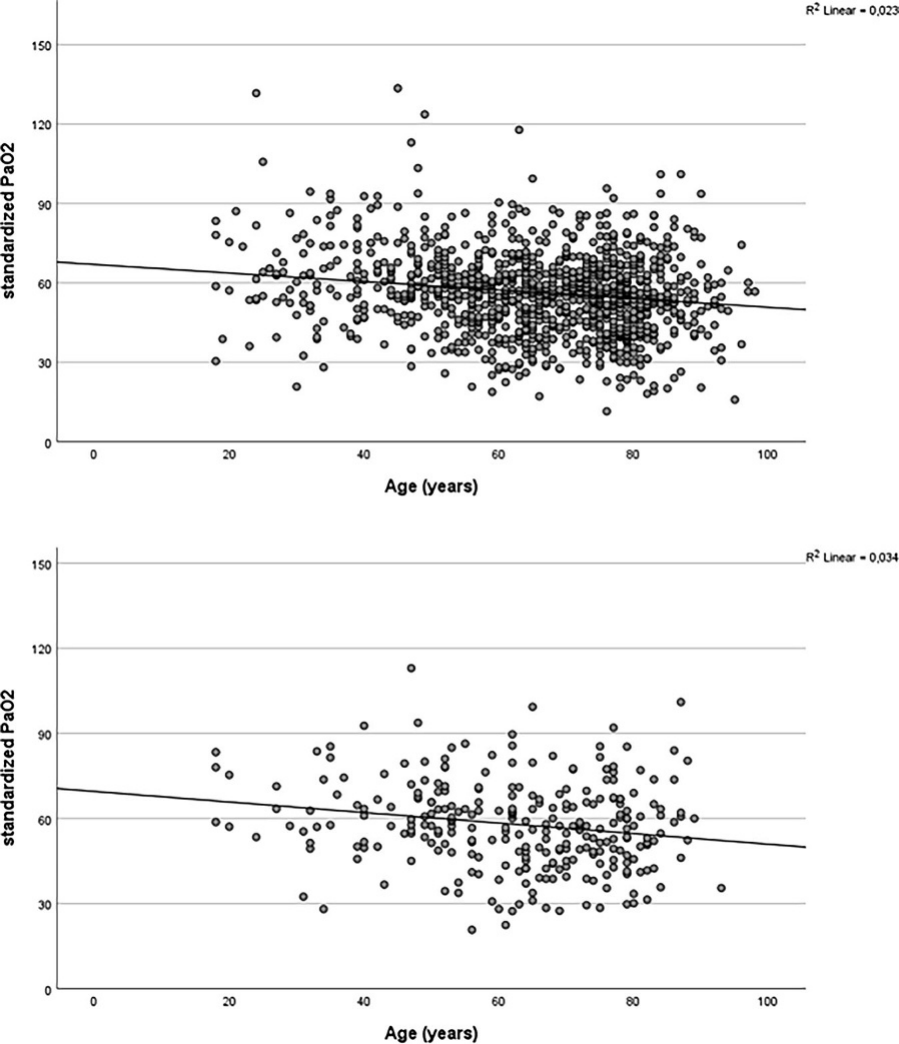
The upper scatter plots illustrate the trends in standardized PaO_2_ in capillary blood gas analysis across the age spectrum in the entire cohort of patients with suspected PE (*n* = 1073). The lower scatter plots demonstrate the standardized PaO_2_ in capillary blood gas analysis with increasing age in the cohort of patients with confirmed PE who met all criteria of the new algorithm (*n* = 278). *PE* pulmonary arterial embolism

For the evaluation of blood gases, all patients with possible or documented oxygen administration at admission (358 of 465 patients) and with an unknown extraction point of the BGA (62 of 465 patients), missing documentation (7 of 465 patients) or venous BGA (41 of 465 patients) were excluded. Based on these criteria, 1073 of 1538 patients could be considered for further analysis. Of these 1073 patients, a total of 278 patients were diagnosed with PE. Table 2 shows the results of the remaining 278 patients. Overall, 243 patients aged < 80 years and 35 patients aged ≥ 80 years could be analyzed with respect to BGA and PTP. There was only a statistically significant difference in the simplified revised Geneva score, which was slightly higher in elderly patients (*p* = 0.042). In addition, the changes in standardized PaO_2_ with increasing age were evaluated in patients with capillary BGA and without administration of oxygen within the entire cohort (*n* = 1073) and in patients with confirmed pulmonary artery embolism (*n* = 278). The results are shown in Fig. 2 and imply a decrease in the linear regression graphs with increasing age.

**Table 2:** Capillary BGA and PTP scores of patients with confirmed PE

	< 80 years (*n* = 243)	≥ 80 years (*n* = 35)	*p*-value
Wells score	1.5 (0, 1.5)	1.5 (0, 3)	*p* = 0.309
Simplified Wells score	1 (0, 1)	1 (0, 1)	*p* = 0.758
Original revised Geneva score	3 (2, 5)	4 (1, 6)	*p* = 0.721
Simplified revised Geneva score	2 (1, 2)	2 (1, 3)	***p*** **=** **0.042**
BGA pH	7.46 (7.44, 7.49)	7.48 (7.45, 7.53)	*p* = 0.087
BGA PaCO_2_	34 (29, 37)	32 (28, 36)	*p* = 0.226
BGA PaO_2_	68 (58, 78)	72 (55, 79)	*p* = 0.766
BGA HCO_3_^−a^	23.95 (21.8, 26.08)	23.9 (21.5, 26.1)	*p* = 0.676
BGA BE^a^	0.9 (−0.65, 2.55)	1.1 (−1.05, 3.48)	*p* = 0.686
BGA lactate^b^	1.0 (0.8, 1.6)	1.1 (0.9, 1.5)	*p* = 0.298
BGA saturation^c^	96.00 (93.35, 97.78)	96 (92.7, 97.2)	*p* = 0.950
PaO_2_stand	57.38 (48.34, 67.06)	53.04 (42.40, 63.36)	*p* = 0.254

Data presented as median (IQR). Numbers in bold type indicate a significant difference

*PE* pulmonary embolism, *PTP* pretest probability, *BGA* blood gas analysis, *PaCO*_2_ arterial carbon dioxide tension, *PaO*_*2*_ arterial oxygen tension, *HCO3−* bicarbonate, *BE* base excess, *PaO*_2_*stand* standardized to a PaCO_2_ of 40 mm Hg [24]

^a^*n* = 271, ^b^*n* = 272, ^c^*n* = 253

### BGA-optimized PTP

**Fig. 3:**
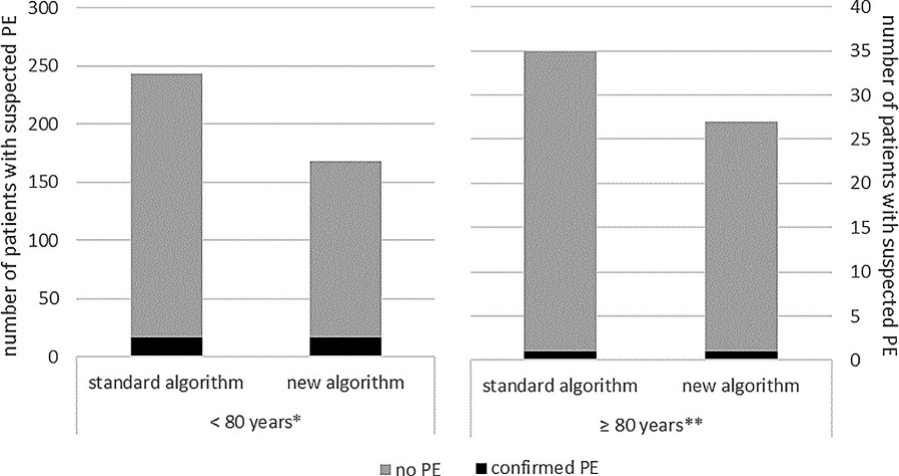
Number of examined patients with low pretest probability analyzed separately according to standard algorithm and novel algorithm. *n = 243, **n = 35. PE was detected in a total of 18 patients (< 80 years: *n* = 17 of 243 patients, ≥ 80 years: *n* = 1 of 35 patients). Using the new algorithm, 33.2% (75 of 226 patients) of all patients < 80 years and 23.5% (8 of 34 patients) of all patients ≥ 80 years would not have been classified as suspected PE. *PE* pulmonary embolism

In a next step, we performed an age-stratified analysis of the previously published algorithm with BGA-optimized PTP. Therefore, we focused on patients with a low PTP (defined by a Wells score ≤ 4), available capillary BGA and (age-adjusted) increased D‑dimers with an upper limit of < 1.5 mg/L. A total of 278 patients met these criteria, and PE was detected in a total of 6.5% (*n* = 18) of these patients (elderly patients: 1 of 35 patients; younger patients: 17 of 243 patients). With application of our new algorithm using a standardized PaO_2_ of 65 mm Hg (Fig. 1), the number of unnecessary CTPA examinations could be reduced by 33.2% in younger patients (75 of 226 patients without PE) and 23.5% in elderly patients (8 of 24 patients without PE, Fig. 3).

## Discussion

With this study we can make an important contribution to an optimized ED management of patients aged ≥ 80 years with suspected or confirmed PE who are currently underrepresented in clinical trials [2]. First, we characterize clinical findings and prognostic parameters in octogenarians with confirmed PE compared to younger patients. Overall, elderly patients with PE are characterized by higher clinical risk markers and elevated mortality rates compared to younger patients. Moreover, to our knowledge, this is the first study to investigate a BGA-optimized Wells score in elderly patients using a standardized PaO_2_ in order to reduce the number of unnecessary CTPAs. With this study, we were able to demonstrate that in patients with suspected PE but low PTP a significant number of unnecessary CTPAs could be avoided by using an BGA-optimized pretest algorithm even in elderly patients.

The comparison of all baseline characteristics reveals that patients aged ≥ 80 years are significantly more likely to be female. This is likely due to the longer life expectancy of female patients, a finding consistent with other studies of very old patients with PE [14]. Gómez et al. [14] found similar results irrespective of the PTP. However, the study also found significant differences in dyspnea, chest pain and hemoptysis, which could not be confirmed by the analysis of our cohort. Nevertheless, these results do not contradict our findings, but rather emphasize the heterogeneity of the clinical presentation of patients with PE [15]. Our trial data also showed that older patients had less tachycardia, more cardiovascular comorbidity and more oxygen administration. This appears plausible in the context of the increasing number of comorbidities with advancing age [16] and the higher number of medications in this patient cohort [17]. Additional documentation of medication upon admission was not conducted in this retrospective study, which would have further strengthened the validity of the results. However, it is not uncommon for elderly patients ≥ 65 years of age to be prescribed β‑blockers [17], which could explain the reduced heart rate observed in older patients compared to younger ones. Furthermore, elderly patients also have a higher risk profile. Patients ≥ 80 years of age were significantly more likely to have signs of right heart dysfunction in CTPA or echocardiography, elevated cardiac markers (troponin and nt-pro-BNP) and a higher sPESI and were, therefore, assigned to a higher risk class. Accordingly, the in-hospital mortality was also significantly higher in these patients, although the significantly different cohort size and the lack of documentation of the cause of death must also be considered when analyzing the data. Study data also show similar or even higher mortality rates within 30 days in elderly patients ≥ 80 years, even after pulmonary artery embolism has been excluded [18]. Overall, these results are consistent with previous studies showing that elderly patients with acute pulmonary embolism have higher in-hospital mortality than younger patients [19].

There is limited data available on very elderly patients with PE. A cut-off of 65 years is often used as a definition for elderly [15, 20], but this threshold needs to be reconsidered in the context of demographic changes [21] and the continuously improving medical care. Additionally, diagnostics are challenging regardless of age [14]. The lack of significant differences in diagnostic and clinical findings between older and younger patients with confirmed PE further emphasizes these difficulties. For example, there was no significant difference in chest pain, dyspnea or ECG documentation when comparing the two age cohorts. Therefore, other parameters like blood gases should be considered. Notably, there was no significant difference in BGA separated by age cohorts. A number of factors appear to be involved in this process. First of all, the group of patients aged ≥ 80 years is relatively small, which must be taken into account during statistical evaluation. Additionally, the age-associated decline in standardized PaO_2_, although not statistically significant, is noticeable. This trend is also illustrated by linear regression slopes in the scatter plot, indicating an impact on the effectiveness of the blood gas-optimized algorithm. Follow-up studies with larger cohorts are necessary to verify this trend.

On the other hand, the absence of a statistical difference in PaO_2_ should be recognized as an indicator for this parameter’s limitations, as it does not account for potential hypocapnic hyperventilation. This limitation is overcome by the use of standardized PaO_2_, i.e., the value of paO_2_ adjusted for partial pressure of carbon dioxide. Standardized PaO_2_ better reflects the respiratory efforts in hypoxemic conditions and may have greater diagnostic significance in the assessment of suspected PE. The reduction in CTPA examinations by using our BGA-optimized algorithm published previously was lower in the present subanalysis. Given that the current PaO_2_ is used to calculate the standardized PaO_2_, and this value declines with increasing age due to physiological changes [22, 23], the significance of using standardized PaO_2_ within our algorithm is diminished. Nonetheless, 23.5% of CTPAs could still be avoided in patients over 80 years, compared to 33.2% in those younger than 80 years. Thus, younger patients particularly benefit from reduced exposure to unnecessary X‑rays. However, avoiding nearly one in four CTPA examinations in this elderly patient cohort still represents a relevant resource-efficient reduction of overall CTPA examination.

### Limitations

Several aspects should be considered when interpreting our results. For example, the retrospective data were only obtained from single center and, therefore, cannot be generalized. In addition, the group of patients over 80 years of age is comparatively small, especially when looking at BGAs. Although this analysis is based on a large and well-characterized cohort of patients with suspected PE, larger prospective studies are needed to validate our proposed score in patients with suspected PE and low PTP. In addition, the cause of death during hospitalization was not recorded. Therefore, it remains unclear whether this was related to PE or another cause. Moreover, patients with lung disease were also included in our study (table S1). These study data, therefore, represent a clinically relevant but also heterogeneous patient population. However, an influence of the pulmonary diseases on the results cannot be excluded with certainty. Further studies with larger numbers of patients are necessary to investigate this aspect. The present study also represents a significant improvement in the management of patients with suspected pulmonary embolism. However, it is important to recognize the potential for selection bias due to the retrospective nature of the data analysis and the initial exclusion process.

## Conclusion

The present study shows that elderly patients with confirmed pulmonary embolism (PE) have a higher clinical risk profile and higher all-cause in-hospital mortality compared to the respective younger cohort. It also demonstrates the additional benefit of a blood gas analysis (BGA)-optimized algorithm including standardized PaO_2_ to improve pretest probability (PTP) assessment even in elderly patients with suspected PE and, thus, reduce the number of computed tomography pulmonary angiography (CTPA) examinations. We therefore suggest implementing the following ideas:

- Routine implementation of a capillary BGA,
- Calculation of the standardized PaO_2_,
- Implementation BGA results in the decision on further diagnostics in combination with the two-level Wells score for suspected PE, and
- Increased awareness for elderly patients with pulmonary artery embolism

## Supplementary Information

Table S1. Pulmonary diseases in patients with confirmed pulmonary embolism sorted by age group.

Table S2. Baseline characteristics and risk stratification of all patients with unknown extraction point of the blood gas analysis (BGA), missing documentation or venous BGA or administration of oxygen (group 1) compared to patients without administration of oxygen and capillary BGA (group 2)

## Data Availability

The raw data supporting the conclusions of this article will be made available by the authors upon reasonable request.

## Declarations

The study was approved by the local ethical committee (reference number: 19-318A).

The supplement containing this article is not sponsored by industry.
