# Supplementary material for: Clinical characteristics and BGA-optimized pretest probability of pulmonary embolism in the elderly
Source: Med Klin Intensivmed Notfmed. 2025 Jan 22;120(Suppl 1):8–14. doi: 10.1007/s00063-024-01235-8 (PMC12708831; doi:10.1007/s00063-024-01235-8)
Supplement: Supplementary file 1 — Table S1. Pulmonary diseases in patients with confirmed pulmonary embolism sorted by age group. [file 63_2024_1235_MOESM1_ESM.docx]

**ONLINE SUPPLEMENTS**

**Table S1. Pulmonary diseases in patients with confirmed pulmonary embolism sorted by age group.**

|  | **<80 years (n=71)** | **≥ 80 years (n=17)** |
| --- | --- | --- |
| chronic obstructive pulmonary disease (COPD) | 20 (28.2%) | 7 (41.2) |
| emphysema | 2 (2.8%) | 4 (23.5%) |
| bronchiectasis | 1 (1.4%) | 1 (5.9%) |
| asthma | 11 (15.5%) | 2 (11.8%) |
| bronchial carcinoma or pulmonary metastases | 20 (28.2%) | 2 (11.8%) |
| diaphragmatic paralysis with restrictive ventilatory impairment | 1 (1.4%) |  |
| ventilatory impairment associated with obstructive sleep apnea | 3 (4.2%) |  |
| pulmonary hypertension | 3 (4.2%) |  |
| idiopathic pulmonary fibrosis | 1 (1.4%) |  |
| hiatal hernia with nearly fully intrathoracic stomach | 1 (1.4%) |  |
| multiple bipulmonary granulomas | 1 (1.4%) |  |
| sarcoidosis with pulmonary involvement | 1 (1.4%) |  |
| cavitation following severe pneumonia | 1 (1.4%) |  |
| unclear pulmonary disease | 5 (7%) | 1 (5.9%) |

Data presented as n/N (%).
