# Supplementary material for: Clinical characteristics and BGA-optimized pretest probability of pulmonary embolism in the elderly
Source: Med Klin Intensivmed Notfmed. 2025 Jan 22;120(Suppl 1):8–14. doi: 10.1007/s00063-024-01235-8 (PMC12708831; doi:10.1007/s00063-024-01235-8)
Supplement: Supplementary file 2 — Table S2. Baseline characteristics and risk stratification of all patients with unknown extraction point of the blood gas analysis (BGA), missing documentation or venous BGA or administration of oxygen (group 1) compared to patients without administration of oxygen and capillary BGA (group 2) [file 63_2024_1235_MOESM2_ESM.docx]

**Table S2. Baseline characteristics and risk stratification of all patients with unknown extraction point of the BGA, missing documentation or venous BGA or administration of oxygen (group 1) compared to patients without administration of oxygen and capillary BGA (group 2)**

|  | **group 1 (n=465)** | **group 2 (n=1073)** | **p-value** |
| --- | --- | --- | --- |
| male | 237 (51) | 525 (48.9) | p=0.463 |
| confirmed PE | 143 (30.8) | 290 (27) | p=0.136 |
| age | 71 (60, 79) | 68 (56, 78) | **p<0.001** |
| central PE | 78 (16.8) | 126 (11.7) | p=**0.008** |
| segmental PE | 47 (10.1) | 136 (12.7) | p=0.153 |
| subsegmental PE | 18 (3.9) | 28 (2.6) | p=0.182 |
| chest pain | 157 (33.8) | 429 (40) | p=**0.021** |
| dyspnea | 335 (72) | 649 (60.5) | **p<0.001** |
| hemoptysis | 22 (4.7) | 41 (3.8) | p=0.408 |
| syncope | 65 (14) | 166 (15.5) | p=0.452 |
| clinical signs of deep vein thrombosis | 63 (13.5) | 178 (16.6) | p=0.132 |
| tachycardia (>100bpm) | 171 (36.8) | 288 (26.8) | **p<0.001** |
| S1Q3 pattern | 50 (10.8) | 92 (8.6) | p=0.175 |
| T wave inversion in V1-V4 | 41 (8.8) | 84 (7.8) | p=0.515 |
| complete/incomplete RBBB | 47 (10.1) | 136 (12.7) | p=0.153 |
| history of cardiovascular disease | 116 (24.9) | 237 (22.1) | p=0.221 |
| history of pulmonary disease | 145 (31.2) | 210 (19.6) | **p<0.001** |
| history of renal disease | 44 (9.5) | 94 (8.8) | p=0.658 |
| active cancer | 93 (20.0) | 178 (16.6) | p=0.107 |
| previous PE or DVT | 56 (12.0) | 158 (14.7) | p=0.163 |
| D-dimer^a^ , mg/L | 2.48 (1.3, 5.48) | 2.05 (1.22, 4.9) | p=**0.027** |
| right heart dysfunction in CTPA or echocardiography^b^ | 110 (30.2) | 161 (17.9) | **p<0.001** |
| elevated Troponin serum concentration ^c^ | 211 (70.1) | 376 (51.4) | **p<0.001** |
| elevated NTpro-BNP serum concentration^d^ | 105 (57.4) | 199 (47.2) | p=**0.021** |
| sPESI |  |  |  |
| EMR: low risk^e^ | 16 (11.2) | 82 (28.4) | **p<0.001** |
| EMR: intermediate low risk^f^ | 56 (38.9) | 131 (45.3) | p=0.202 |
| EMR: intermediate high risk^f^ | 59 (41) | 69 (23.9) | **p<0.001** |
| EMR: high risk^f^ | 13 (9.0) | 5 (1.7) | **p<0.001** |
| confirmed DVT^g^ | 75 (48.7) | 169 (48.1) | p=0.909 |
| In-hospital death^h^ | 8 (5.4) | 8 (2.8) | p=0.158 |

Data presented as n/N (%) or median (IQR). Numbers in bold type indicate a significant difference. PE: pulmonary embolism, DVT: deep vein thrombosis, RBBB: right bundle branch block, BGA: blood gas analysis, CTPA: computed tomography pulmonary angiography, sPESI: simplified PESI (Pulmonary Embolism Severity Index), EMR: early mortality risk of in-hospital or 30-day death*.* a: n=1419, b: n=1265, c: n=1032*,* d: n=605, e: n=432, f: n=433, g: n=505, h: n=437
